# Supplementary figures and images for: Prevalence and prediction of Lyme disease in Hainan province
Source: PLoS Negl Trop Dis. 2021 Mar 18;15(3):e0009158. doi: 10.1371/journal.pntd.0009158 (PMC8009380; doi:10.1371/journal.pntd.0009158)

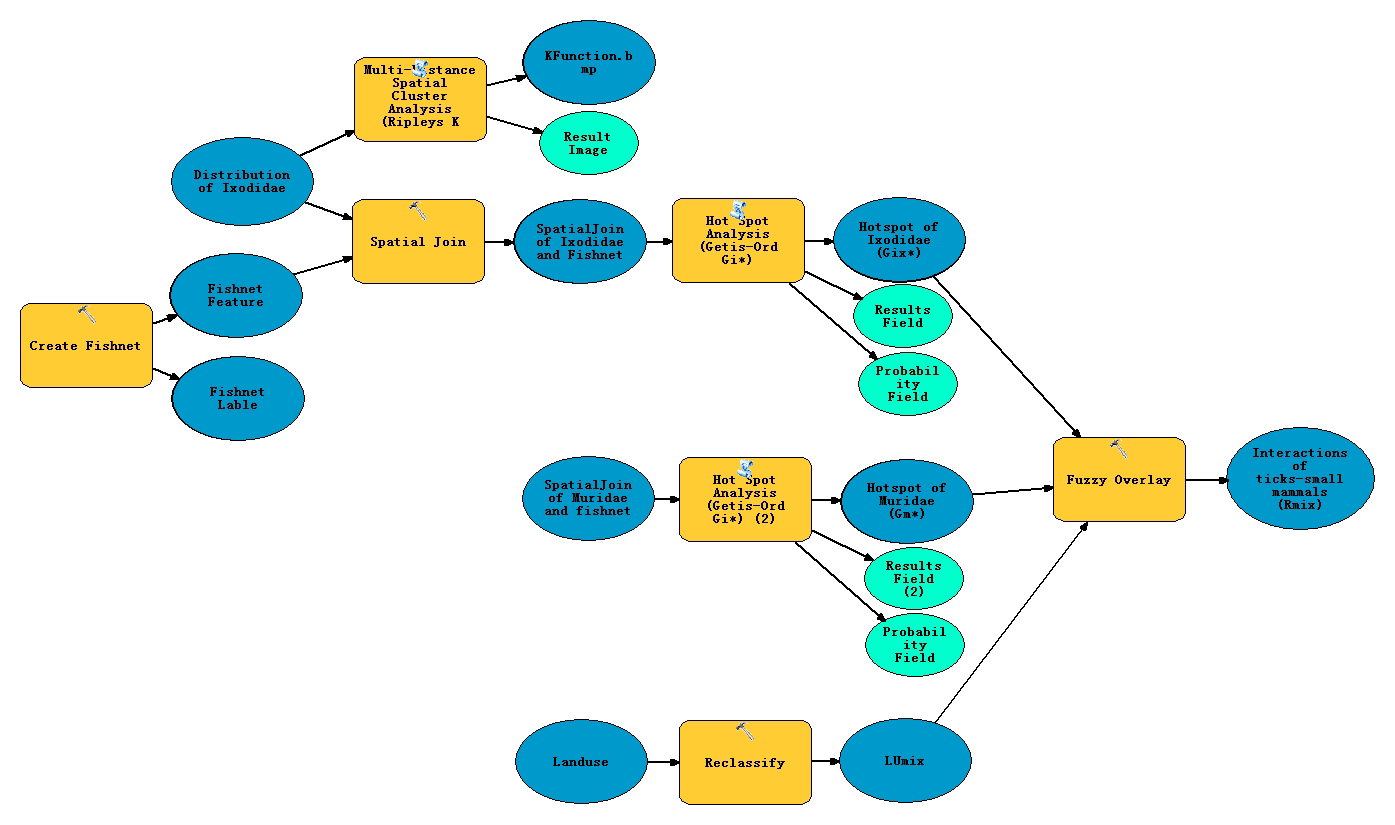

Supplement: S1 Fig — (TIF) [file pntd.0009158.s001.tif]

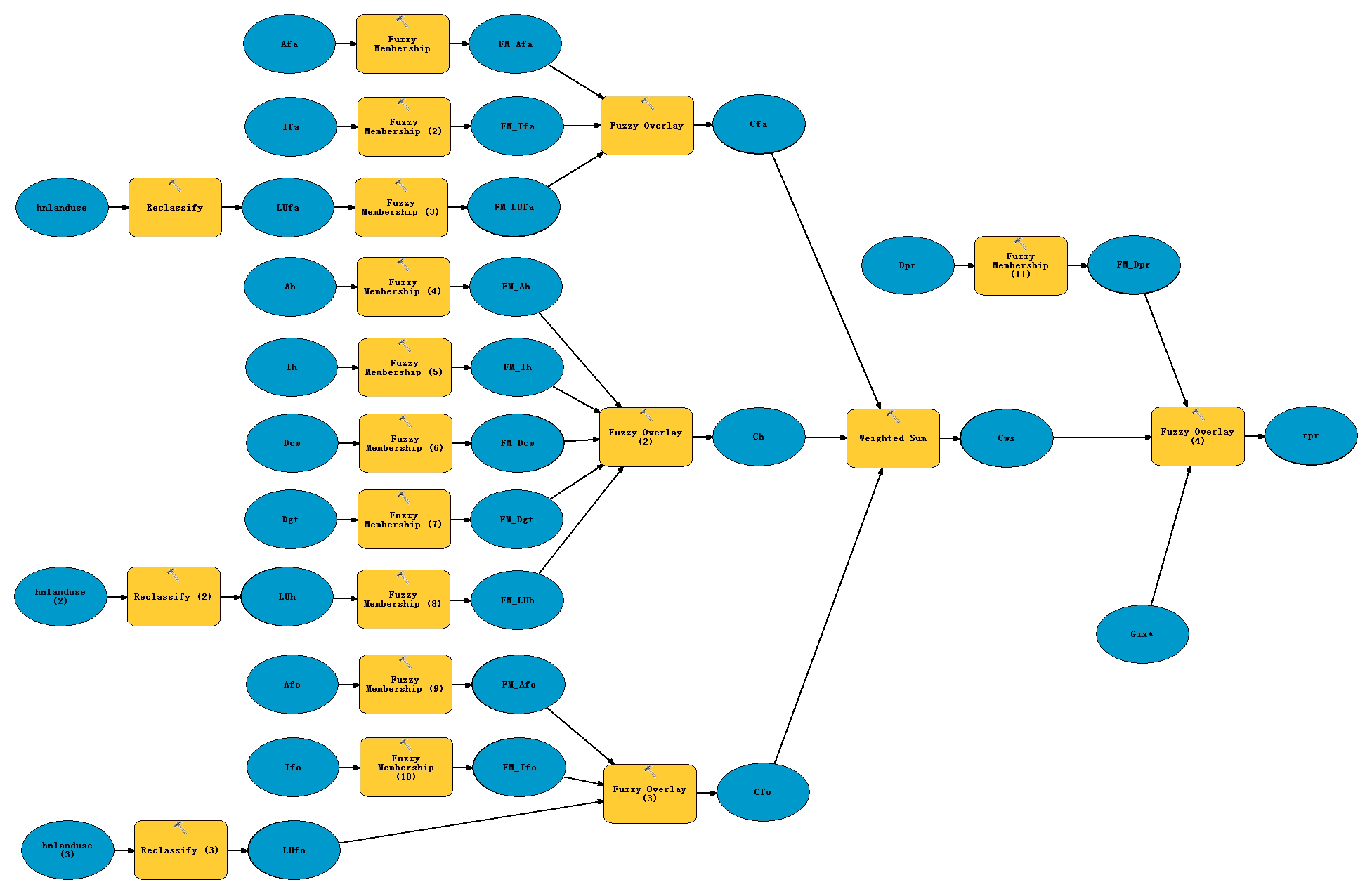

Supplement: S2 Fig — (TIF) [file pntd.0009158.s002.tif]

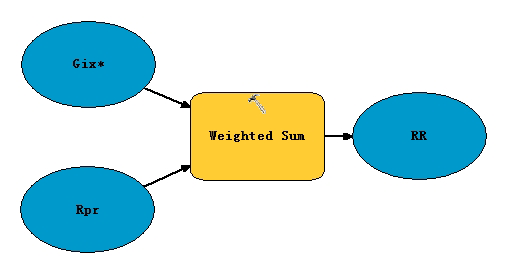

Supplement: S3 Fig — (TIF) [file pntd.0009158.s003.tif]
