# Supplementary material for: Prevalence and prediction of Lyme disease in Hainan province
Source: PLoS Negl Trop Dis. 2021 Mar 18;15(3):e0009158. doi: 10.1371/journal.pntd.0009158 (PMC8009380; doi:10.1371/journal.pntd.0009158)
Supplement: S1 Table — (DOC) [file pntd.0009158.s004.doc]

Land Cover data attribute table

| Primary category | | Secondary category | |
| --- | --- | --- | --- |
| Value | Name | Value | Name |
| 1 | Cultivated land | 11 | Rice paddy field |
|  |  | 12 | Dry land |
| 2 | Forest land | 21 | Forest |
|  |  | 22 | bush |
|  |  | 23 | Sparse woods |
|  |  | 24 | Others |
| 3 | grassland | 31 | High coverage grassland |
|  |  | 32 | Medium coverage grassland |
|  |  | 33 | Low coverage grassland |
| 4 | Wet land | 41 | River |
|  |  | 42 | Lake |
|  |  | 43 | Reservoir |
|  |  | 45 | Seaside wetland |
|  |  | 46 | Bottomland |
| 5 | Urban, rural area and construction land | 51 | Urban |
|  |  | 52 | Rural area |
|  |  | 53 | Construction land |
| 6 | Unused land | 61 | Sand |
|  |  | 63 | Saline and alkaline land |
|  |  | 64 | Marshland |
| 9 | Sea | 99 | Sea |

The land cover data were derived from the 2020 Landsat8 Land Cover product, Land Cover Type Yearly National 1km, which classifies land cover as 6 primary categories and 25 secondary categories.

**Reclassification remap table of Land Cover for Land** use

| Old Values | Count | Class Name | New Values |
| --- | --- | --- | --- |
| 11 | 3075 | Rice paddy field | 1 |
| 12 | 5573 | Dry land | 2 |
| 21 | 13006 | Forest | 6 |
| 22 | 2417 | bush | 5 |
| 23 | 942 | Sparse woods | 6 |
| 24 | 5098 | Others | 6 |
| 31 | 996 | High coverage grassland | 4 |
| 32 | 167 | Medium coverage grassland | 4 |
| 33 | 15 | Low coverage grassland | 4 |
| 41 | 184 | River | 7 |
| 42 | 49 | Lake | 7 |
| 43 | 968 | Reservoir | 7 |
| 45 | 142 | Seaside wetland | 7 |
| 46 | 100 | Bottomland | 7 |
| 51 | 366 | Urban | 8 |
| 52 | 547 | Rural area | 3 |
| 53 | 481 | Construction land | 8 |
| 61 | 75 | Sand | 9 |
| 63 | 1 | Saline and alkaline land | 9 |
| 64 | 16 | Marshland | 9 |
| 99 | 69 | Sea | 10 |

Land cover types were combined and reclassified into 10 land use categories, which were rice paddy field, dry land, grassland, bush, forest land, wet land, rural area, urban and construction land, unused land and sea.
