# Supplementary material for: Prevalence and prediction of Lyme disease in Hainan province
Source: PLoS Negl Trop Dis. 2021 Mar 18;15(3):e0009158. doi: 10.1371/journal.pntd.0009158 (PMC8009380; doi:10.1371/journal.pntd.0009158)
Supplement: S2 Table — (DOC) [file pntd.0009158.s005.doc]

**Reclassification remap table of Land-use layer for *LUmix*.**

| Old Values | Count | Class Name | New Values |
| --- | --- | --- | --- |
| 1 | 3075 | Rice paddy field | 6 |
| 2 | 5573 | Dry land | 7 |
| 3 | 547 | Rural area | 5 |
| 4 | 1178 | Grassland | 8 |
| 5 | 2417 | Bush | 9 |
| 6 | 19046 | Forest land | 4 |
| 7 | 1443 | Wet land | 1 |
| 8 | 847 | Urban and construction land | 3 |
| 9 | 92 | Unused land | 2 |
| 10 | 69 | sea | 1 |

We generated $LUmix$ layer to demonstrate the area of Muridae and Ixodidae contact. We reclassified the land-use layer which the possible contact area: bush as “9”, grassland as “8”, dry land as “7”, rice paddy field as “6”; the rare contact area: rural area as “5”, forest land as “4”, urban and construction land as “3”, unused land as “2”, while we assigned grid value of impossible contact area-lake and sea -as “1”.

**Reclassification remap table of Land-use layer for *LUfa*.**

| Old Values | Count | Class Name | New Values |
| --- | --- | --- | --- |
| 1 | 3075 | Rice paddy field | 4 |
| 2 | 5573 | Dry land | 12 |
| 3 | 547 | Rural area | 12 |
| 4 | 1178 | Grassland | 1 |
| 5 | 2417 | Bush | 1 |
| 6 | 19046 | Forest land | 1 |
| 7 | 1443 | Wet land | 1 |
| 8 | 847 | Urban and construction land | 1 |
| 9 | 92 | Unused land | 1 |
| 10 | 69 | sea | 1 |

In order to display where the farming activity happens on map, we generated raster of farmland (LUfa) using reclassify tool in ArcGIS. As 68% rice paddy fields were three ripe cycle land (wet-wet-dry) in Hainan, also based on 8-hour working/about 40 minutes time spent on the way, we assigned the value of the dryland and rural area grids to “12”, whereas rice paddy field grids were “4” and other land-use type grids were “1”.

**Reclassification remap table of Land-use layer for *LUh*.**

| Old Values | Count | Class Name | New Values |
| --- | --- | --- | --- |
| 1 | 3075 | Rice paddy field | 1 |
| 2 | 5573 | Dry land | 1 |
| 3 | 547 | Rural area | 12 |
| 4 | 1178 | Grassland | 12 |
| 5 | 2417 | Bush | 12 |
| 6 | 19046 | Forest land | 1 |
| 7 | 1443 | Wet land | 1 |
| 8 | 847 | Urban and construction land | 1 |
| 9 | 92 | Unused land | 1 |
| 10 | 69 | sea | 1 |

We created raster of grassland and bush (*LUh*) layer, where grassland, bush and rural area grids were reclassified as “12” and other grids were “1”.

**Reclassification remap table of Land-use layer for *LUfo*.**

| Old Values | Count | Class Name | New Values |
| --- | --- | --- | --- |
| 1 | 3075 | Rice paddy field | 1 |
| 2 | 5573 | Dry land | 1 |
| 3 | 547 | Rural area | 12 |
| 4 | 1178 | Grassland | 1 |
| 5 | 2417 | Bush | 12 |
| 6 | 19046 | Forest land | 12 |
| 7 | 1443 | Wet land | 1 |
| 8 | 847 | Urban and construction land | 1 |
| 9 | 92 | Unused land | 1 |
| 10 | 69 | sea | 1 |

The raster of forests(*LUfo*) layer was the reclassified land-use layer where forest land, bush and rural area grids value were assigned as “12” and other grids were “1”.
