# Supplementary material for: Prevalence and prediction of Lyme disease in Hainan province
Source: PLoS Negl Trop Dis. 2021 Mar 18;15(3):e0009158. doi: 10.1371/journal.pntd.0009158 (PMC8009380; doi:10.1371/journal.pntd.0009158)
Supplement: S3 Table — (DOC) [file pntd.0009158.s006.doc]

The Distance Band using Multi-Distance Spatial Cluster Analysis tool (Ripleys K) with Muridae

| Field1 | ExpectedK | ObservedK | DiffK |
| --- | --- | --- | --- |
| 0 | 0.04877777500000 | 0.29765960971222 | 0.24888183471222 |
| 0 | 0.09755555000000 | 0.30912180391038 | 0.21156625391038 |
| 0 | 0.14633332500000 | 0.32135232900324 | 0.17501900400324 |
| 0 | 0.19511110000000 | 0.33131371201937  0.34800650185777 | 0.13620261201937 |
| 0 | 0.24388887500000 |  | 0.10411762685778 |
| 0 | 0.29266665000000 | 0.36497158547307 | 0.07230493547307 |
| 0 | 0.34144442500000 | 0.39520393552048 | 0.05375951052048 |
| 0 | 0.39022220000000 | 0.40540198610317 | 0.01517978610318 |
| 0 | 0.43899997500000 | 0.42470886073375 | -0.01429111430000 |
| 0 | 0.48777775000000 | 0.44436790957609 | -0.04340984040000 |

The Distance Band using Multi-Distance Spatial Cluster Analysis tool (Ripleys K) with Ixodidae

| Field1 | ExpectedK | ObservedK | DiffK |
| --- | --- | --- | --- |
| 0 | 0.04411600000000 | 0.41928209274477 | 0.37516609274477 |
| 0 | 0.08823200000000 | 0.41928209274477 | 0.33105009274477 |
| 0 | 0.13234800000000 | 0.41928209274477 | 0.28693409274477 |
| 0 | 0.17646400000000 | 0.41928209274477 | 0.24281809274477 |
| 0 | 0.22058000000000 | 0.41928209274477 | 0.19870209274477 |
| 0 | 0.26469600000000 | 0.42528643789440 | 0.16059043789440 |
| 0 | 0.30881200000000 | 0.42528643789440 | 0.11647443789440 |
| 0 | 0.35292800000000 | 0.42528643789440 | 0.07235843789440 |
| 0 | 0.39704400000000 | 0.46333115658249 | 0.06628715658249 |
| 0 | 0.44116000000000 | 0.47236380286899 | 0.03120380286899 |
