# Supplementary material for: Prevalence and prediction of Lyme disease in Hainan province
Source: PLoS Negl Trop Dis. 2021 Mar 18;15(3):e0009158. doi: 10.1371/journal.pntd.0009158 (PMC8009380; doi:10.1371/journal.pntd.0009158)
Supplement: Attachment — Submitted filename: review.pdf [file pntd.0009158.s011.pdf]

Check numerous typos and extensive grammar errors throughout.

Software and risk factors

paragraph 1: More needs to be said about "all data were entered into ArcGIS as raster layers." One nearly never creates raster layers from scratch. Were they derived from satellite imagery? Converted from vector data? What does "entered" mean here?

paragraph 2: The authors likely mean "land cover" instead of "land use." The source of the land cover data should be provided, as should a source or method of generating the "tick species distribution" layer. A description of what went into the "Tick Active Area" and "Tick Risk Area" layers is needed, as is some rationale supporting the reclassification into 9 classes. Why, for example, do the authors consider farmland a greater risk than bush? Why does seaside wetland appear to be rated higher risk than forest? It is probably not necessary to list the number of pixels containing each land cover type.

paragraph 3: "As tick questing ask for a certain height." is a typo? Last sentence is virtually a duplicate of the last sentence of paragraph 2 and is unnecessary.

Paragraph 4: The reclassification scheme could be better conveyed using a remap table. What made the authors decide that "more tick species could increase the risk of lyme disease" – as opposed to greater tick population or greater chance of human exposure? Should be supported with citation to past research if this is true.

Analyses

This section needs considerable expansion, perhaps to include any meaningful spatial analysis at all. How was the Tabulate Area output used and interpreted? What were the grounds for deciding that those were high risk areas? What was the means of inference to other areas? Are "cities" the polygonal regions shown in the map?

Figure 1

This is certainly not a map of 1583 individuals. There are about 200 points in the map, and they are not well-distributed throughout the island. Perhaps the large point symbols obscure multiple individuals, in which case a smaller symbol should be used. Also some reference to the underlying population distribution would be helpful. Why are the areas with nearly no points so sparse – does no one live there? Also a description of the process used to geocode addresses is needed.

Figure 2 – A

Because of sampling bias, this map could easily be interpreted merely as a map of which districts have a testing hospital in them. The legend is badly done. It is misleading to show 6 decimal places on numbers with so little precision. The legend should be more explicit as to the quantity represented by the color ramp (it's IFA positive rate). Why map this instead of the 2-step positives?

Patient Distribution and Risk Analysis

Paragraph 2: "*According to the survey, there were 12 species ticks in Qiongzhong (Supplemental material, S1 Table), which is the area with most tick species in Hainan.*" According to the S1 Table,

there were also 12 tick species in Ledong and 10 in Sanya, both of which have no positive cases (and nearly no samples). How do the authors account for these counter examples to their argument (probably sampling bias)? Would they expect high values here if they could now acquire samples? Perhaps that would be way to test their theory.

"*In all 5 areas in our research, the spread of Lyme diseases were basically matched with the tick distribution.*" The word "spread" implies an expansion over time, which is unsupported by the rest of the work. A map of the tick distribution (the number of species present in each district?) might help.

Figure 3

There does not seem to be much predictive about Figure 3. We see the same 9 classes described above . . . are we just seeing land cover with numeric labels? If not, what went into this index? Is it an average of the rather arbitrary scores assigned to land cover, the reclassified number of tick species per district and . . . . what? Still don't know at this point what "Tick Active Area" and "Tick Risk" are.

Nothing in the paper supports (or even describes) the drawing of those arrows, as if the authors intend to show past or future change in the spatial pattern of the disease.

Discussion

paragraph 6: Similarly, there is nothing to support the verb "radiated" implying spread over time "*The risk of lyme disease in Hainan was radiated from Wuzhi Mountain in the east, south and west directions . . .* "

"*In the northwest of Wuzhi Mountain, there is Limu Mountain which hampered the distribution of lyme disease.* " If mountain barriers are this effective, perhaps they should be included in the risk index.
