# Supplementary material for: Prevalence and prediction of Lyme disease in Hainan province
Source: PLoS Negl Trop Dis. 2021 Mar 18;15(3):e0009158. doi: 10.1371/journal.pntd.0009158 (PMC8009380; doi:10.1371/journal.pntd.0009158)
Supplement: Attachment — Submitted filename: Responses.doc [file pntd.0009158.s012.doc]

Dear Editor，

Thank you for your help and patience. We have made some responses to the reviewers’ comments below.

We have tried our best to do the revision. We hope that it will work.

Thanks for all that you’ve done for us.

Sincerely,

Lin.

About Methods:

Reviewer #2: The authors need explain carefully on how you linked hospital data to your environmental predictors, which is very important to evaluate the performance of your predictive models. The present methods only focused much on the process of testing the disease in the lab. What is even more important is to provide the steps linking these test data with environmental climatic niches and human activities, which are unfortunately lacking here.
Risk factors
There is no any fundamental information on how you selected the 17 cities as your study sample areas. The explicated geographical positions and occurrence of the Lyme disease reporting should also be provided here.
The authors need explain the reason why you chose the three factors here. In addition to human activities and host characteristics, fundamental niches such as climate and microhabitat factors determine the suitability of the disease survival (e.g., Guernier et al. 2004, Liu et al. 2013). Just as what I have suggested on the Introduction section, the authors need strengthen the hypotheses around disease prevalence.
Ref.
Guernier V, Hochberg ME, Guegan JFO. (2004) Ecology drives the worldwide distribution of human diseases. PLoS Biol. 2, 740–746.
Liu X, Rohr JR, Li Y (2013) Climate, vegetation, introduced hosts and trade shape a global wildlife pandemic. Proceedings of the Royal Society B: Biological Sciences, 280: 20122506.
“were no data…” should be revised to “included no data…”
[27] red color?
One major issue in the Data analysis and Result sections is that the authors did not provide any statistical analyses to evaluate the performance of your predictive model. Although the GIS spatial analysis is fine with me, there are at least some approaches such as randomization test to validate the robustness of your predictor selection and overall predictive power of your model.
Fig. 3 please add the place name in the map to facilitate the understanding of spatial distributions of Lyme hotspots.

Our responses:

1, The serums were from patients who were neurological or arthritis patients. While, these data were not overall investigation in whole Hainan province. Therefore, they can not reflect the integrated disease infection geographically and statistically. Instead, we visualized the distribution of confirmed lyme patients and compared with our risk map. These results could reflect correlation of the disease distribution and risk prediction directly.

2, In our analysis, rural human density, cow density, goats density, total value of farming, husbandry and forestry (from the Statistical Yearbook) were county-level data. While, vector and host data were point data with longitude and latitude, and these data were plotted as a point layer using Display XY Data Tool in ArcGIS. Then the landuse data was raster layer. The way of plotting the geographical positions of the tested Lyme disease case was like vector and host data.

3, Instead of the last version of those three factors (potential vector distribution layer, number of vector species and landuse layer), we thoroughly considered the interaction among host, vector and human. Therefore, we added the hotspots of Muridae and Ixodidae to indicate the interaction between host and vector; and rural human density, farming activity, husbandry activity, forestry activity and hotspots of Ixodidae, etc to map the risk from interction between human and vector. Finally, we calculated the sum of these two risks to indicate Lyme risk in Hainan.

In a year, climate and microhabitat factors could bave deep implications for the disease survival. While in our analysis,we want to show that the distribution of the risk based on the knowledge and information.

4, Our prediction method was knowledge based analysis, in which all data we used were proved important in lyme disease.

5, We added the place name in the map.

Rusuts:

Reviewer #2: I suggest shorting the paragraph on Lyme test, which is not related with your main model analysis. By contrast, the authors should pay more attentions to clarify the relationship between your hospital test and modeling analyses.
Weather should be whether
The paragraph of “as we know” is weak in logic and utility, which should be modified using more clear hypotheses driving the outbreak and prevalence of the Lyme disease. The present writing did not provide readers enough information on the background of factors important to Lyme prevalence in your study area, China and other regions worldwide.
It is better to introduce a series of abiotic, biotic and human activities in determining the disease prevalence. This information is very helpful to represent the three predictors used in the following GIS models. In addition, the authors need strengthen the distributional patterns and crucial predictors of disease in a general way (i.e., not specific to the Lyme but other related neglected tropical bacterial diseases worldwide before talking about the Lyme disease.
As the disease widely distributes across the mainland of China, the following paragraph should stress more on the important implication of conducting Lyme disease risk analyses in Hainan province of China. I am not able to follow the present description only addressing the unique geographical position and climate. The authors need explain more on the unique opportunity of conducting this research in Hainan province, China. Only the lack of related study in Hainan is not a reasonable ground of a novel study. Furthermore, the authors need to provide readers some background knowledge on the differences of disease prevalence between mainland and island, which will make the present work more interesting and general.

Our responses:

1, Suggestions Accepted.

2, Our prediction also could be used for other tick-borne diseases, but the interaction among related factors should be reorganized according to the disease distribution and prevalence pattern.

3, The important of research on Lyme disease should be addressed.

Conclusions:

Reviewer #1: I'd suggest being explicit about the limitations.

Reviewer #2: The present conclusion is too descriptive and should be strengthened by more important implications based on their main findings.

Reviewer #3: More ambitious conclusions are claimed than can be supported by the data and analysis (see Summary & General Comments response).

Our responses:

1, We added the limitations.

2, Suggestion accepted.

3, Out conclusions were changed base on results.

Editorial and Data Presentation Modifications?

Reviewer #1: See summary and general comments below

Reviewer #2: I have no specific comments on this field.

Reviewer #3: Significant grammar and spelling errors throughout.

Out response:

We have made the revision about grammar and spelling errors.

Summary and General Comments:

• Abstract: significant language issues

Our response:

We rewrite the Abstract. We changed “Lyme disease is one of the most important neglected tropical bacterial diseases” as “Lyme disease (LD) is one of the most important vector-borne diseases worldwide.” Spelling errors had been corrected.

• How were the hospitals selected? Include brief description of the size and scope of those hospitals. Similarly, it is important to understand the representativeness of the sampling approach. How was the sample size determined?

Our response:

The 5 hospitals were all the representative hospital in local area. We added this information to the manuscript.

• Need more detail about the selection/inclusion criteria i.e. “arthritis and nervous system disease”? How long had they been symptomatic? Any further clinical detail e.g. history of rash? Duration of symptoms also impacts interpretation of the results

Our response:

Lyme disease is a systemic, tick-borne disease with protean manifestations, including dermatologic, rheumatologic, neurologic, and cardiac abnormalities. The best clinical marker for the disease is the initial skin lesion, erythema migrans. While in China, except Dongbei Forest area and Inner Mongolia Forest area, lyme patients with erythema migrans were rare in investigation or report, or it may because the investigation was insufficient. Some patients may delay the illness to late manifestations because Lyme disease was neglected in China. Lyme arthritis and Nervous system are common late manifestations. Antibodies tests of Late Lyme patients would not lead to misdiagnose for they already passed antibodies testing negative stage. Therefore, in our analysis, we only test serum antibodies with two-tier tests, but not polymerase chain reaction.

• Provide more information on local tick vectors and reservoir hosts

Our response:

We added tick vectors and reservoir hosts in our analysis. (see Methods)

• Add a limitations section

Our response:

We added the limitations section. (see discussion)

• Could provide a few sentences on the clinical/public health implications of Lyme disease, if left neglected

Our response:

Research of LD in Hainan was since 2005 and still was neglected for hospitals in Hainan still cannot do the lyme test. Clinical test of Lyme disease could tell how many Lyme patients were there in Hainan and what were their distribution.

• Try to contextualize the prevalence findings by offering a few sentences on the regional and/or international estimates

Our response:

There were Lyme diseases in all 5 areas in our investigation. Lyme disease mostly distributed in temperate zone, was tested prevalent in tropical area in China. Clinicians should pay more attention of Lyme disease in this area.

• Is there evidence of other tick-borne illnesses in Hainan? Have other agents been investigated locally and/or in China e.g. Babesia, anaplasma, Borrelia miyamotoi?

Our response:

Tick-borne disease spotted fever was firstly reported since 1990s(see Introduction). After that, there were some investigations about tick and there hosts. However, there was still lack of the systematic and overall research about vector-borne diseases.

References:

Liu JH, Yu XL, Long ZM, et al. The investigation of spotted fever in Hainan Island. J Prevent Med PLA. 1996;14: 317-319.

Huang JL, Zhou PS, Long ZM. Community structure and population spatial distribution of parasitic ticks, mites and fleas in Hainan mountainous regions. *Entomol Knowledge.* 1997;34:337-339.

Sun DW, Wang SQ, Zhao Wei, et al. Seasonal succession of ticks in small wild animals in Chengmai county of Hainan province. *Chin J Public Health.* 2012;28:1620-1622.

Jin YM, Zhang LJ, Sun LY, Lao SJ, Han J, et al. Epidemiological investigation of emerging spotted fever in Chengmai county, Hainan province. *Dis Surveil.* 2011;26:18-22

Chang BG, Zhang JZ, Tian XD, et al. Study on seroepidemiological investigation of spotted fever in Qiongzhong Area of Hainan province. *Chin J Vector Bio & Control.* 1999;10:198-200.

Tian XD, Chen WJ, Lu Z, et al. The investigation on host and vector of spotted fever in Dafeng farm of Qiongzhong in Hainan province. *Chin J Zoonoses.* 1999;15:94.

• Resolution of Fig 1 map needs to be improved

Our response:

OK.

Tables and Figures
• Figures need legends

Our response:
OK.

• Supplementary table needs a legend and explanation

Our response:

OK.

Reviewer #2: Zhang et al. used 1,583 serum samples across 5 hospitals of Hainan province, China to explore the prevalence and potential predictors of Lyme disease. The topic fits well within the scope of PNTD, and the sampling effort at a province scale is impressive. In addition, Hainan province is very unique in its biodiversity and biogeography in China, and thus understanding the prevalence and the risks of important neglected tropical bacterial diseases is very timely and crucial for the prevention and control of the disease. However, my major concerns are that the current manuscript is too descriptive and should be strengthened especially for the hypothesis and additional data analyses to validate the performance of your predictive models. In addition, the scientific writing could be improved with an aid of a native speaker.

Our response:

Many thanks.

For the analysis, we reanalyzed the prediction of Lyme disease using knowledge-based method. We rewrite the whole article.

Reviewer #3: Software and risk factors
paragraph 1: More needs to be said about “all data were entered into ArcGIS as raster layers.” One almost never creates raster layers from scratch. Were they derived from satellite imagery? Converted from vector data? What does “entered” mean here?

Our response:

Data on rural people density, cow density, goat density and intensity of farming activity, husbandry, forestry, etc., collected from the Hainan Yearbook, were county-level data. These data were entered at a new field in attribute table of Hainan county vector layer in ArcGIS.

paragraph 2: The authors likely mean “land cover” instead of “land use.” The source of the land cover data should be provided, as should a source or method of generating the “tick species distribution” layer. A description of what went into the “Tick Active Area” and “Tick Risk Area” layers is needed, as is some rationale supporting the reclassification into 9 classes. Why, for example, do the authors consider farmland a greater risk than bush? Why does seaside wetland appear to be rated higher risk than forest? It is probably not necessary to list the number of pixels containing each land cover type.

Our response:

Landuse layer was from Wikipedia. We abandoned the “tick species distribution” layer. Instead, we generated the hotspots of ticks using tick distribution (see Analysis ).

paragraph 3: “As tick questing ask for a certain height.” is a typo? Last sentence is virtually a duplicate of the last sentence of paragraph 2 and is unnecessary.

Our response:

We rewrite the whole Method part.

Paragraph 4: The reclassification scheme could be better conveyed using a remap table. What made the authors decide that “more tick species could increase the risk of lyme disease” – as opposed to greater tick population or greater chance of human exposure? Should be supported with citation to past research if this is true.

Our response:

In revised version of our manuscript, we only reclassified the landuse layer to map where farming, husbandry and forestry located. We will add remap table in Supporting information captions.

Analyses
This section needs considerable expansion, perhaps to include any meaningful spatial analysis at all. How was the Tabulate Area output used and interpreted? What were the grounds for deciding that those were high risk areas? What was the means of inference to other areas? Are “cities” the polygonal regions shown in the map?

Our response:

We reanalyzed the risk of Lyme disease from the interaction of host-vector and human-vector. Lyme disease as a vector borne disease was determined by the whole ecosystem, which include the environment, interaction between host and vector, interaction between human and vector, and so on. Therefore, risk of Lyme disease was not limited by the city boundary, but ecosystem and human interference.

Figure 1
This is certainly not a map of 1583 individuals. There are about 200 points in the map, and they are not well-distributed throughout the island. Perhaps the large point symbols obscure multiple individuals, in which case a smaller symbol should be used. Also some reference to the underlying population distribution would be helpful. Why are the areas with nearly no points so sparse – does no one live there? Also a description of the process used to geocode addresses is needed.

Our response:

Points in this map seem less than 1583, that’s true, because locations of patients were from their addresses. A lot of the addresses were not specific to street or door. Instead, some of these were village or town. Therefore, a lot of points in the map contain more than one patients. While, this is the map of 1583 individuals. And they are not well-distributed throughout the island, because we only investigate the lyme disease in 5 counties in Hainan for some reason.

Figure 2 – A
Because of sampling bias, this map could easily be interpreted merely as a map of which districts have a testing hospital in them. The legend is badly done. It is misleading to show 6 decimal places on numbers with so little precision. The legend should be more explicit as to the quantity represented by the color ramp (it’s the IFA positive rate). Why map this instead of the 2-step positives?

Our response:

We modified the legend. As IFA positive rate would reflect the infection of Lyme disease in one place, that is to say, high IFA positive means more prevalent. And Figure2-B was the 2-step positives, which shows the distribution of the confirmed patients.

Patient Distribution and Risk Analysis
Paragraph 2: “According to the survey, there were 12 species ticks in Qiongzhong (Supplemental material, S1 Table ), which is the area with most tick species in Hainan.” According to the S1 Table, there were also 12 tick species in Ledong and 10 in Sanya, both of which have no positive cases (and nearly no samples). How do the authors account for these counter examples to their argument (probably sampling bias)? Would they expect high values here if they could now acquire samples? Perhaps that would be way to test their theory.
“In all 5 areas in our research, the spread of Lyme diseases were basically matched with the tick distribution.” The word “spread” implies an expansion over time, which is unsupported by the rest of the work. A map of the tick distribution (the number of species present in each district?) might help.

Our response:

Thank you very much for these ideas. We have changed this part in manuscript.

Discussion
paragraph 6: Similarly, there is nothing to support the verb “radiated” implying spread over time “The risk of lyme disease in Hainan was radiated from Wuzhi Mountain in the east, south and west directions . . . ”
“In the northwest of Wuzhi Mountain, there is Limu Mountain which hampered the distribution of lyme disease. “ If mountain barriers are this effective, perhaps they should be included in the risk index.

Our response:

That’s true we can not conclude that “risk radiated...” from our results. We have already changed this part.
