# Supplementary material for: Prevalence and prediction of Lyme disease in Hainan province
Source: PLoS Negl Trop Dis. 2021 Mar 18;15(3):e0009158. doi: 10.1371/journal.pntd.0009158 (PMC8009380; doi:10.1371/journal.pntd.0009158)
Supplement: Attachment — Submitted filename: Response Letter.doc [file pntd.0009158.s013.doc]

Dear Editor，

Thank you for your help and patience. We have made some responses to the reviewers’ comments below.

We have tried our best to do the revision. We hope that it will work.

Thanks for all that you’ve done for us.

Sincerely,

Lin.

**About Methods:**

Reviewer #2: The revised method section is much more improved. Especially it has been much clearly for the part of spatial analyses. My final suggestion is that the authors need explain a bit more on the screening process of the host distribution data from GBIF. GBIF database includes occurrences in suspicious locations (e.g., on the grounds of a museum, the epicenter of a city, etc.), which are needed to remove. These records are usually incomplete sightings/specimens that are re-labelled later by people who don’t know where the sighting/collection took place.  
**Our responses:** There were two geographical distribution data (in all 72 distribution spots from GBIF in Hainan) of Muridae, which were *Rattus tanezumi* and *Chiropodomys gliroides*. These two genera were on the grounds of a museum. We put *Rattus tanezumi* into our analysis, as: 1) this data was valuable as there was lack of investigation in small-mammals in Hainan; 2) *Rattus tanezumi* as some pathogens’ natural host was wildly distributed all over China mainland and Hainan province [1]. Whereas, *Chiropodomys gliroides* was reported spreading in South Asia long time ago, but was not found in China nowadays. Therefore, we removed this data in our manuscript.

Reviewer #3: The revised analysis is more sound than the original, but some methodological ambiguities and weaknesses remain. The objectives are articulated and the study design is appropriate, but details of the execution may lead to inaccurate results.  
-- One flaw remaining in the spatial analysis portion of the revised paper is the source of the land use data. The authors cite Wikipedia and claim it was downloaded from there. I am unable to find any land use raster at the link provided, and Wikipedia is generally not in the business of doing land use classification of satellite imagery. If the authors found a dataset there, it must have come with a reference to a primary source. This matters for two reasons: for confidence in the risk analysis, it can be helpful to know how the land uses were derived and what categories were contemplated, and it is essential to know the cell size of the original to evaluate how much resampling was needed in the 10km grid analysis (for example in the computation of Cfa, Ch, and Cfo). Land use is usually derived from land cover, and categories like “sparse woods, seaside wet lands, slope grassland” sound more like land cover. The authors might consider a description of the land use /  
land cover data (but applicable to whatever they in fact used) like “The land use data were derived from the 2020 MODIS Terra+Aqua Combined Land Cover product, Land Cover Type Yearly L3 Global 500m, June 2020, which classifies land cover according to the International Geosphere-Biosphere Programme 17-class scheme. Land cover types were combined and reclassified into [some number of] land use categories following [some citation to the work used to inform their land use classification].”  
**Our response:** We reanalyzed the model with new land cover data, which were derived from Resource and Environment Science and Data Center (<http://www.resdc.cn/data.aspx?DATAID=335>). These data contains 6 primary categories and 25 secondary categories. In order to show where the contacts between small mammals and ticks or human beings and ticks, we reclassified the land cover types into 10 categories, which were rice paddy field, dry land, grass land, bush, forest land, wet land, rural area, city or construction land, unused land and sea.

— The separate components of the LUmix layer are clear enough, but include the method of their final combination into one layer. A map of the LUmix layer would be good to include, as it is an important component of the spatial analysis.

**Our response:** In our analysis, LUmix demonstrated the potential contact area of Muridae and Ixodidae. Our final combination was to integrate the risk from host-tick and human-tick interactions. As in previous analysis in those two risk assessments have already include the reclassified landuse layer to identify where those interactions happened. Therefore, there is no need to combine landuse layer again in the final combination.

— The authors make a somewhat light argument in passing for the use of a 10km grid for the rodents (searching for food), but they don’t defend its use for ticks. Is it a reasonable grid size on which to see tick clustering and habitat extent? Some supporting citation to ecological literature on gridding species ranges would be valuable here.

**Our response:** As previous study suggested that host movements contribute importantly to tick distribution, especially in lyme disease.[2-4] Therefore, we used 10km grid for ticks as well.

— The hotspot analysis seems fairly sound, but the authors might explain what hot spots/Getis G contributes that their original spatial join count of observations to grid cells does not. Why is the degree of spatial clustering more informative than just high incidence in this case? Similarly, they should explain why Fuzzy Membership is used instead of, say, simply converting to z-scores for commensurability.

**Our response:** We have already added the explanations in the manuscript. We mapped the hot spot area for Muridae or tick distribution in Hainan using Getis-Ord Gi* instead of original spatial join counts, as the investigation of these animals were scattered data points as presence/absence data which could not thoroughly reflect their possible distribution considering spatial autocorrelation. We need to model the risk areas of their potential distribution hot spots, spatial clustering may show the appropriate area for these animals. Therefore, we used hot spot instead of high incidence accumulation. The spatial join tool only makes the presence data displayed in the fishnet feature.

While we did the “Lyme Risk from host-tick interactions” analysis, we wanted to overlay the Gm*, Gix* and LUmix layers, which had different dimensions. In ArcGIS, Fuzzy Membership tool reclassifies or transforms the input data to a 0 to 1 scale. Combination of Fuzzy Membership and Fuzzy Overlay tool could solve this problem.

-- It would be more informative to the user to identify maximum DiffK value produced by the Ripley’s K analysis, maybe even reproduce the output table. Some explication of the meaning or interpretation of the Getis-Ord statistic, beyond the basic formula, would be useful. Also cite the ESRI documentation from which this section derives, and perhaps include the output so the reader can determine if statistically significant clusters were found at this resolution. The authors appear to be using a particular (but valid) special case of the Getis-Ord Hotspot Analysis, in which the spatial clustering is solely location-based, not weighted by the distribution of any particular attribute. Therefore, "where xj is the attribute value of Muridae" is probably not accurate. “[H]ow you construct the Analysis Field determines the types of questions you can ask. Are you most interested in determining where you have lots of incidents, or where high/low values for a particular attribute  
cluster spatially? If so, run Hot Spot Analysis on the raw values or raw incident counts. . . . Alternatively (or in addition), you may be interested in locating areas with unexpectedly high values in relation to some other variable. “<http://resources.esri.com/help/9.3/ArcGISEngine/java/Gp_ToolRef/Spatial_Statistics_tools/how_hot_spot_analysis_colon_getis_ord_gi_star_spatial_statistics_works.htm>

**Our responses:** We added the DiffK Table into the manuscript as S6 Doc. And we did the revisions in the manuscript.

Using the Spatial Join tool, we added the Muridae (Ixodidae) distribution points to the fishnet feature. If the fishnet feature cell j with more presence of Muridae or Ixodidae, we hypothesized that it was more suitable for the animals. Then, we did the hot spot analysis with the presence counts. We actually predicted the potential hot spot of Muridae (Ixodidae) distribution clusters. The data in GBIF database were the presence data of animals but not the density. For this reason, we could not use the raw data in our analysis, otherwise could lead to insufficient results

— It is essential to run the Ripley’s K and Hotspot Analysis in a local projected coordinate system. The authors should disclose the projection in which they conducted their analysis.

**Our responses:** We used GCS_WGS_1984 coordinate system, and this has been added in the manuscript.

— Rmixi =Gm*Gix*LUmix, The authors should consider and explain the effect of negative values and zeroes in this multiplication formula. What happens when Gm is close to -1 (rodent dispersion, not clustering) and Gix is close to -1 (tick dispersion)? A misleading high positive value is produced. (On review of the supporting material, it looks like the authors used Fuzzy Overlay to make this combination, but (1) why? and (2) which? Fuzzy Product sounds problematic in the ESRI documentation and (3) how would Fuzzy Overlay handle the concern about negative values?)

**Our responses:** Negative values were originally generated from the Getis-Ord Gi-star analysis, which means cold spot clusters of Muridae or Ixodidae distributed in the map. We focused on the appropriate area for Muridae or Ixodidae as more rodents and tick might increase the risk of Lyme disases. We intended to demonstrate the hotspots of the rodents and ticks respectively. Overlapping the hotspots of Muridae and Ixodidae, and land cover layer based on our knowledge, we wanted to assess the Lyme risk from host-tick interactions. While the insignificant cold spots of these two genera, as well as dispersion values were both the low risk area of Lyme disease. This might due to the area actually are low risk with Lyme disease, or for the reason of the knowledge limitation.

We used fuzzy overlay tool as Gm, Gix and LUmix have different dimensions. Direct overlay with these layers would lead to the unbelievable and inexplicable results. Fuzzy membership normalized the data layer, but could not change the meaning of the data, and so was fuzzy overlay. That is to say the unimportant negative values (cold spot clusters of animals) still were irrelevant to the risk of Lyme disease.

— The authors should consult and cite some resources on designing risk analysis formulae.

**Our responses:** References have been added in the manuscript.

— A source is needed for the human population density raster. Also consider that the most densely populated areas (urban) are probably at the least risk.

**Our responses:** Despite with the high dense population, the urban areas were at least risk of lyme disease. As there were less wild mammals as disease reservoirs and ticks as vector to transmit the spirochetes from animals to human. The separation of wild life and human are likely to cut off the transmission route of Lyme disease. In our risk analysis, we only considered the possible transmission route, like small mammals-ticks route, rural people-ticks route during work time. Most importantly, we put more focus on the high risk of Lyme disease. Therefore, we only used rural population density raster in human-tick interaction analysis.

— “Dpri is risk from small mamal-tick interactions within the ith cell” This seems like a typo. Dpr was population density, and Rpr was a risk index that didn’t involve the mammals (rodent).

**Our response:** That was a typo. Thanks for your serious review.

-- It would be interesting, but probably not essential, to include some sort of model validation, formalize how or whether the predicted risk aligned with the discovered cases.

**Our responses:** We will investigate the proper and useful way to make model validation with Lyme prevalence data. More importantly, our model not just a model which could be used with other diseases or in other areas, but also provide a scientific reference which would be a guidance with local Lyme disease investigation. Meanwhile, more investigations would test the model in return.

**Results:**

Reviewer #3: The figures are NOT of sufficient quality for clarity. Values in map legends and labels on maps are too blurry to read.

**Our response:** We have remapped the figures with resolution 300 dpi in TIFF form.

**Conclusions:**

Reviewer #3: The conclusions are improved in scope. They appropriately describe the risk of encounter and its relationship to the observed incidence of disease. If the methodology can be strengthened to give better confidence in the derived results, this should be an informative paper.

**Our response:** Thanks for your advice.

**Editorial and Data Presentation Modifications?**

Reviewer #3: The paper is still rife with English grammar errors and needs to be proofread.  
"In rural area, people get to contact with ticks most likely happened when farming, husbandry or forestry activities go through."  
It's also possible that some of the more grammatical and stylistically unique sentences are taken verbatim from other works and should be encased in quotation marks, e.g.  
"For persistence of lyme borreliosis in the field, a seasonal synchronicity among the questing stages of vectors and the abundance of reservoir hosts is also necessary."

**Our response:** We have made the revisions in the manuscript.

**Summary and General Comments:**

Reviewer #3: I don't think new experiments are needed per se, just some serious strengthening of the execution and documentation of the existing ones. Perhaps the authors might reconsider their multiplicative risk indices based on further review of the literature.

**Our response:** Based on our knowledge, there are insufficient data on dealing with risk indices in LD research. We will keep on exploring the methods which would provide insights of diseases distribution spatially, and even real-time and effective risk assessment of the disease.

References:

1. Guo, S., Li, G., Liu, J. *et al.* Dispersal route of the Asian house rat (*Rattus tanezumi*) on mainland China: insights from microsatellite and mitochondrial DNA. *BMC Genet* **20,** 11 (2019). https://doi.org/10.1186/s12863-019-0714-3.

2. Ostfeld RS, Glass GE, Keesing F. Spatial epidemiology: an emerging (or re-emerging) discipline. Trends Ecol Evol. 2005 Jun;20(6):328-36. doi: 10.1016/j.tree.2005.03.009. PMID: 16701389.

3. Das, Abhik & Lele, Subhash & Glass, Gregory & Shields, Timothy & Patz, Jonathan. (2002). Modeling a discrete spatial response using generalized linear mixed models: Application to Lyme disease vectors. International Journal of Geographical Information Science. 16. 151-166. 10.1080/13658810110099134.

4. Estrada-Peña, Agustín. (2003). The relationships between habitat topology, critical scales of connectivity and tick abundance Ixodes ricinus in a heterogeneous landscape in northern Spain. Ecography. 26. 661-671. 10.1034/j.1600-0587.2003.03530.x.
